# Negative pressure of the environmental air in the cleaning area of the materials and sterilization center: a systematic review

**DOI:** 10.1590/1518-8345.1140.2781

**Published:** 2016-09-01

**Authors:** Caroline Lopes Ciofi-Silva, Lisbeth Lima Hansen, Alda Graciele Claudio dos Santos Almeida, Julia Yaeko Kawagoe, Maria Clara Padoveze, Kazuko Uchikawa Graziano

**Affiliations:** 1 Doctoral Student, Escola de Enfermagem, Universidade de São Paulo, São Paulo, SP, Brazil.; 2 Doctoral Student, Escola de Enfermagem, Universidade de São Paulo, São Paulo, SP, Brazil. Professor, Universidade Federal do Amazonas, Manaus, AM, Brazil.; 3 PhD, Professor, Faculdade Ciências da Saúde Albert Einstein, São Paulo, SP Brazil. RN, Hospital Israelita Albert Einstein, São Paulo, SP, Brazil.; 4 PhD, Professor, Escola de Enfermagem, Universidade de São Paulo, São Paulo, SP, Brazil.; 5 PhD, Full Professor, Escola de Enfermagem, Universidade de São Paulo, São Paulo, SP, Brazil.

**Keywords:** Aerosols, Sterilization, Air Pressure

## Abstract

**Objective:**

to analyze the scientific evidence on aerosols generated during cleaning activities of health products in the Central Service Department (CSD) and the impact of the negative pressure of the ambient air in the cleaning area to control the dispersion of aerosols to adjacent areas.

**Method:**

for this literature systematic review the following searches were done: search guidelines, manuals or national and international technical standards given by experts; search in the portal and databases PubMed, Scopus, CINAHL and Web of Science; and a manual search of scientific articles.

**Results:**

the five technical documents reviewed recommend that the CSD cleaning area should have a negative differential ambient air pressure, but scientific articles on the impact of this intervention were not found. The four articles included talked about aerosols formed after the use of a ultrasonic cleaner (an increased in the contamination especially during use) and pressurized water jet (formation of smaller aerosols 5μm). In a study, the aerosols formed from contaminated the hot tap water with *Legionella pneumophila* were evaluated.

**Conclusions:**

there is evidence of aerosol formation during cleanup activities in CSD. Studies on occupational diseases of respiratory origin of workers who work in CSD should be performed.

## Introduction

Aerosols are generated and released by humans in various activities, such as breathing, talking, coughing and sneezing; Bathing with contaminated water; aerosolization of sewage waste in toilets or drainage system for outdoor environments; cleaning and rinsing surfaces indoors; spraying in agriculture^1^. Aerosols are defined as smaller particles or equal to 5μm, that may or may not contain an infectious agent and, due to their size, can remain suspended in the atmosphere for hours, slowly being transported over long distances and achieve adjacent areas^2^.

Aerosols containing an infectious agent that remain in the environment can be inhaled by susceptible individuals, even if there is no close contact with the disposing source, or contaminated surfaces^3^. The main diseases transmitted by aerosols are tuberculosis, measles and chicken pox. However, there are reports of aerosolization of other microorganisms such as fungi, *Clostridium difficile* and *Staphilococcus aureus* ^4^ ^-^ ^5^.

The droplets that are larger than 5μm, remain suspended for a few seconds and quickly lay on the floor or other surfaces due to gravity. Its liquid portion can evaporate, depending on the environmental conditions, resulting in aerosols. There are slight variations of the nomenclature and definition of sizes, however the Brazilian National Health Surveillance Agency (ANVISA) uses the definition that aerosols are smaller than 5μm, which was adopted for this study^2^ ^-^ ^3^. 

Ventilation systems and air conditioning in various establishments promote comfort, and are useful in the prevention and treatment of diseases transmitted via aerosols. The use of these systems in health services (HS) requires special attention. The basic differences stem from the need to restrict the dispersion of air within an environment to adjacent areas; the specific requirements for ventilation and filtration aiming to dilute and eliminate contamination; the different requirements of temperature and humidity for each area; and sophistication that is demanded for the project^6^.

Among hospital sectors that require air pressure control, temperature and humidity we highlight the Central Service Department (CSD). The CSD is responsible for medical device (MD) from one use to another, it must contain a reception and cleaning room; a preparation and sterilization room; a chemical disinfection room (where applicable); a monitoring area of the sterilization process and a storage and distribution of sterile materials room^7^. 

The RDC Resolution 15, of March 15, 2012 from ANVISA, disposes on the good practice requirements for the processing of MD and other measures. According to this resolution, the CSD class II (which processes complex materials) and processing companies must maintain a negative differential air pressure between the cleaning area and adjacent areas^7^. The areas adjacent to the reception and cleaning room consist mainly of the preparation and sterilization room of MD and circulation areas of other professionals.

Differential ambient air pressure means that there is a difference in measuring the relative air pressure between two areas. This parameter works to provide a positive or negative pressure within a particular area in order to prevent air from migrating from one to the other. If a room has a negative air pressure it means that the air supply is less than the exhaustion^6^.

With the growing concern for the safety of patients and health professionals, there is a need for implementation of best practices that should be based on proven scientific evidence. Thus, the objective of this systematic literature review was to analyze the scientific evidence for the formation of aerosols during the MD cleaning activities in CSD and the impact of negative air pressure, or to the safety of the material to be sterilized, and for health professionals in the adjacent areas too.

## Method 

The steps of this systematic review followed the guidelines published in the *Preferred Reporting Items for Systematic Reviews and Meta-analysis* (PRISMA) Statement, which aims to help the authors to carry out complete and clear records of a systematic review and meta-analysis^8^.

It was defined as the guiding question of this review: Formation of aerosols occurs during cleaning activities of MD, and what is the impact of negative air pressure in the dispersion of these to adjacent areas?

Inclusion criteria were defined as the PICO strategy as follows: P (Patients) = health professionals or MD cleaning professionals; I (Intervention) = negative pressure; C (Comparison) = normal atmospheric pressure; O (Outcome) = dispersion of aerosols. After an initial search in major databases and query in the manuals and guidelines, we identified the lack of studies, both experimental and field, published specifically regarding the CSD. Therefore, it was defined as inclusion criteria: a. Studies published in full, regardless of the publication date and language; b. Clinical studies, experimental studies, guidelines, manuals and national and international technical standards; c. Studies that reported data on the justification for the presence of negative differential ambient air pressure in MD cleaning area; d. Studies that have addressed the formation of aerosols or droplets during the MD cleaning procedures. Exclusion criteria were defined as: a. Reviews of unsystematic or editorial literature; b. Studies where there was only review of the dispersion aerosols; c. Studies that evaluated other air treatment modes.

Initially a consultation of experts in the field of air treatment methods in Health System was held with the clinical engineers and professionals from the Brazilian Association of Refrigeration, Air Conditioning and Heating - (ABRAVA in Portuguese). These experts were asked about the scientific evidence of the need for negative differential ambient air pressure in CSD, which indicated guidelines, manuals and national and international technical standards for consultation. These documents were accessed through the websites of organizations such as the National Health Surveillance Agency (ANVISA), *Centers for Disease Control and Prevention* (CDC) and the *Association for the Advancement of Medical Instrumentation* (AAMI). The documents that were not available on the Internet were obtained from the documentation center of ABRAVA, such as technical standards and booklets of: Brazilian Association of Technical Standards (ABNT), *American Society of Heating, Refrigerating and Air-Conditioning Engineers* (ASHRAE) and the *American Institute of Architects* (AIA). 

Initially, to search for scientific articles, consultations were held on portal descriptors encyclopedias (*MeSH - Medical Subject Headings* do PUBMED) and ("CINAHL Titles" - *Cumulative Index to Nursing and Allied Health Literature)*, to identify descriptors related to the inclusion criteria. However, to ensure access to all potentially eligible studies, keywords were also used. Therefore, the following descriptors were selected: *equipment and supplies*; *health personnel*; *ventilation*; *air pressure*; *aerosols*; *atmospheric pressure*. And the keywords: *devices*; *health worker*; *negative pressure*; *HVAC system* (*heating ventilation and air conditioning*); *bioaerosol*; *airborne*; *droplet*; *droplet nuclei*. 

As a search strategy, the website and databases were consulted as follows: PUBMED and SCOPUS - the controlled descriptors of MESH were primarily used in a single search box. In CINAHL - through the feature "CINAHL Titles" preferably with the expanded definition of the term, with the search first being carried out with all titles separately and later combining the titles using the Boolean operators. In the *Web of Science* - the controlled descriptors of MESH and synonym keywords were used. Access to Embase was restricted in the country at the time of data collection. Filters were not used in the bases and the Boolean AND operator was used to cross the data between the descriptors of the PICO strategy. The OR operator was used to descriptors or synonyms keywords. The search was carried out between March and April 2015, and updated in June of the same year. The search strategies were built with the help of a librarian expert in database.

Given the scarcity of scientific articles detected initially, it was decided to conduct a comprehensive search, a large number of articles being found with crossings. The titles and summaries of them were read, identifying potentially eligible articles. Thus, 68 articles were read in full by two of the authors of this review, individually, which evaluated whether the articles corresponded to the established inclusion criteria. If there were no correlation, the authors recorded the reason for the exclusion. A third researcher was consulted when there were doubts and disagreements. 

The manual search step was the consultation of all technical document references (when mentioned), of the included articles and non-systematic reviews read in its entirety in order to identify other potentially eligible studies.

The articles selected according to the inclusion criteria were analyzed separately by two reviewers. After the critical reading, reviewers completed a table, prepared by two of the authors of this review in Microsoft Excel, considering the concerned matter. The table was composed of the following items: identification of the article; objective, study outline; study site; aerosolizing mechanism; presence or absence of negative air pressure environment; type of statistical analysis; main results; main conclusions; recommendations for their practice; and finally limitations of the studies. Because of gaps in the methodological descriptions and results, it was necessary to consult a third reviewer to discuss the doubts of all articles. After establishing a consensus among reviewers, a final table with all the relevant data extracted from the studies was prepared.

The risk of bias was assessed according to the design of each study. Due to the heterogeneity of the articles, it was decided to present the results descriptively. Since laboratory experimental studies are not included in the reference classification of evidence, studies of this review were classified as inconclusive, partly conclusive and conclusive, considering the guiding question of this review.

## Results

**Figure 1:**
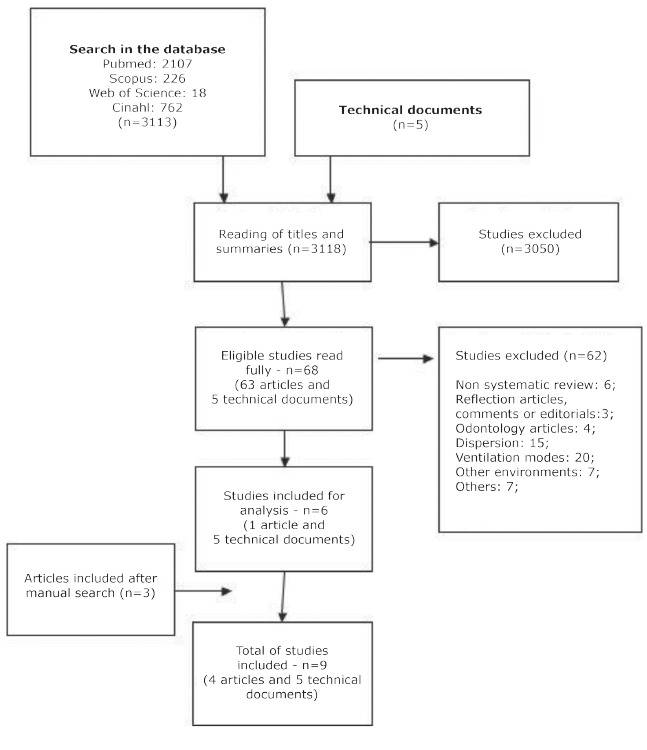
Flowchart of search results strategies, São Paulo, SP, 2015

Five technical documents and four scientific articles that met the inclusion criteria were analyzed. The results of the search strategies are shown in the flowchart (Figure 1).

The experts consulted indicated five technical documents to be analyzed: 2011 ASHRAE *Handbook. Heating, Ventilating and Air Conditioning Application* ^6^; ABNT NBR 7256:2005^9^; *Comprehensive guide to steam sterilization and sterility assurance in health care facilities* from AAMI^10^; *Guidelines for Environmental Infection Control in Health-Care Facilities* from CDC^11^; e *Guidelines for design and construction of hospital and health care facilities* from AIA^12^. 

In the technical standard NBR 7256, of the Brazilian Association of Technical Standards (ABNT/2005)^9^, regarding the air treatment in health care facilities - Requirements for the project and construction of facilities, the environments are classified according to the risk of adverse health events by exposure to ambient air, with numerical assignments, level zero being considered low risk and level three the ambient with high-risk health problems related to air quality. The CSD was classified as level one "an area where it was not found the risk of health problems related to air quality, but some authorities, organizations and researchers suggest that the risk should be considered."

All the technical documents analyzed^6^ ^,^ ^9^ ^-^ ^12^ advocate that the reception area, and the cleaning and separation of CSD materials should have a negative differentiation ambient air pressure relative to adjacent areas (min 2,5Pa), without recirculation of air and that all the air inside should be eliminated directly outside. There is no consensus on the number of air changes per hour, as well as on the dilution method for the elimination of pathogens.

**Figure 2:**
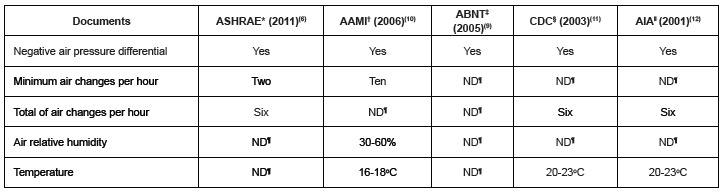
Recommendation of the atmospheric pressure area cleaning Central Service Department (CSD) in relation to the underlying spaces and minimum and total number of exchanges with external air per hour, by year of publication, São Paulo, SP, 2015.

In Brazil, the NBR 7256/2005^9^ is followed, referring to CSD in the cleaning area, which resembles the international recommendations, as shown in Figure 2.

*ASHRAE: *American Society of Heating, Refrigerating and Air-Conditioning Engineers*; †AAMI: *Association for the Advancement of Medical Instrumentation*; ‡ABNT: Brazilian Association of Technical Standards; §CDC: *Centers for Disease Control and Prevention* (CDC); ||AIA: *American Institute of Architects*; ¶ND: undefined

The four scientific articles included^13^ ^-^ ^16^ are studies in English: three^13^ ^-^ ^15^ published between the decades of 1960-1980 and only one in the 2000s^16^; three^13^ ^-^ ^14^ ^,^ ^16^ are laboratory experimental studies and one is transversal^15^ (held in hospital bathrooms); three^13^ ^-^ ^15^ studies conducted in the United States and one in Australia^16^. No studies that evaluated the negative air pressure in MD cleaning areas were found on the searches. Therefore, studies that evaluated the formation of aerosols during cleaning products or surfaces were included, even if not specifically made in a CSD environment.

In three studies^13^ ^-^ ^14^ ^,^ ^16^, experiments were performed to analyze the aerosol recovery generated from equipment commonly used in the CSD: ultrasonic cleaner and scrubbers with pressurized water.

In the study conducted with the ultrasonic cleaner^14^, air samples above the water surface in the washer tank were collected (the air gatherer had a flexible termination that was placed inside the washing machine), aiming to recover aerosols with *Pseudomonas aeruginosa*. Air specimens were obtained in four moments: before the washer was turned on (during 15 minutes), considering that the instruments were placed within it (five minutes), during its operation (25 minutes), and after it was off (15 minutes); covered with a lid (suggesting that all generated aerosols were from the washer); and with the lid off (ambient air added to the aerosol generated by the washer). Samples from surfaces on nine points around the washer (before and after collecting air samples) and from the cleaning solution (10ml) were collected. By analyzing the four moments of air collection, higher average of colonies per ft3 were found during the operation of the washer. And when compared to the results of the samples with and without a lid, statistically significant differences were found, with larger numbers of colonies related to the operation with the lid, which means aerosols formed exclusively by the operation of the washer. The results of surface samples were not influenced by the aerosol, because there was no correlation between the number of colonies found before and after the operation of the washing machine. The authors suggest that surface contamination is related to cleaning solution leakage and dripping during the insertion and removal of the MD, regardless of the washer being with or without the lid. There was no apparent correlation between the contamination of the air and the cleaning solution. The authors indicated that the ultrasonic cleaner should be used with the lid to minimize the release of aerosols to the environment.

Regarding the formation of aerosols from automatic washers with pressurized water, in a study^13^, surface cleaning was performed (wall) with an intentionally contaminated prepared solution of organic food components added with microorganisms (*Serratia marcescens*; spores of *Bacillus subtilis, Staphylococcus aureus* positive coagulase, *Mycobacterium smegmatis* and a bacteriophage virus). The size of the aerosols generated after the use of a pressurized automatic washer and cleaning with stiff bristle brush were compared. In addition, we evaluated the number of viable microorganisms after cleaning only with water and a disinfectant solution (sodium hypochlorite, benzalkonium chloride, peracetic acid, detergents and phenolic disinfectants, non-toxic neutral liquid detergent). The wall was infected one day prior to the collection of air samples, that occurred immediately before, during and after the spraying of the cleaning solution / disinfection, through two types of air collectors: *Andersen sampler* six stages (size evaluation of the particles) and May air sampler (*impingers* - quantitative evaluation). It was observed that after using the washer with pressurized water and the collection with the *Andersen sampler* for 15 to 30 seconds, the number of colonies in the aerosol was greater compared to brushing (24,565 and 1628, respectively). Furthermore, 45.5% of the particles formed after the washer with pressurized water correspond to the third and sixth stages of the gatherer (up to 6μm) and after brushing, 27.6%; therefore, aerosols generated by the washer were smaller. For all the pathogens, the number of viable microorganisms per liter of air collected were higher during the cleaning procedures, when compared to the numbers after completion. When compared with washing only with water, all the cleaning solutions / disinfection reduced the number of viable microorganisms in aerosols and the peracetic acid solution was proved to be more effective. Comparative analyzes using statistical tests were not performed.

In another study^16^, in order to evaluate the formation of aerosols during household activities, a car cleaning experiment was conducted in a controlled environment, sealed with plastic. Two ways of using the hose with pressurized water were tested: spraying (used for rinsing) and a water jet with controlled flow (used to remove dirt), conventional (low pressurization - manual trigger) and efficient (high pressurization). The authors used three gauges of particle sizes not aiming to identify microorganisms in aerosols. It was observed that when used in higher pressurization (efficient method), more and smaller particles were identified (up to 2μm). However, there were no statistically significant differences between the methods: efficient and conventional or between the spray modes and jet. The authors reported that they observed the formation of visible fog in the tent where the experiments were carried out, especially after the high pressurization method, and cogitated they were hydrated aerosols, possibly lower than 500ηm.

A study^15^ was included considering the possibility that the CSD could be equipped with hot tap water. In this one, the formation of aerosols from showers and hot tap water contaminated with *Legionella pneumophila* was evaluated. Samples were collected for cultures of water from showers and faucets; *Swabs* of the internal surface of those; air samples outside the scope of the shower jet; and samples of 14 air rooms, where the gatherer was positioned 61cm away from the tap area. Two types of air collectors were used: *Andersen sampler* six stages (Calibrated to collect particles 0,65μm to 3,3μm in stages four, five and six and in stages one, two and three particles larger than 3,3μm) and two stages (differentiation only in two particle sizes: 0.8 to 8μm and greater than or equal to 8μm). Air samples were collected before the tap was opened, while open, and after being closed. Of 19 paired samples of tap water or *swab* of the tap's nozzle and air samples, in 17 water samples and in 13 air samples there was identification of bacteria colonies. Eleven samples with positive cultures were obtained while the tap was open. Aerosols generated by taps were fewer in number and larger in size when compared to aerosols of the showers. No statistical tests were applied. 

**Figure 3:**
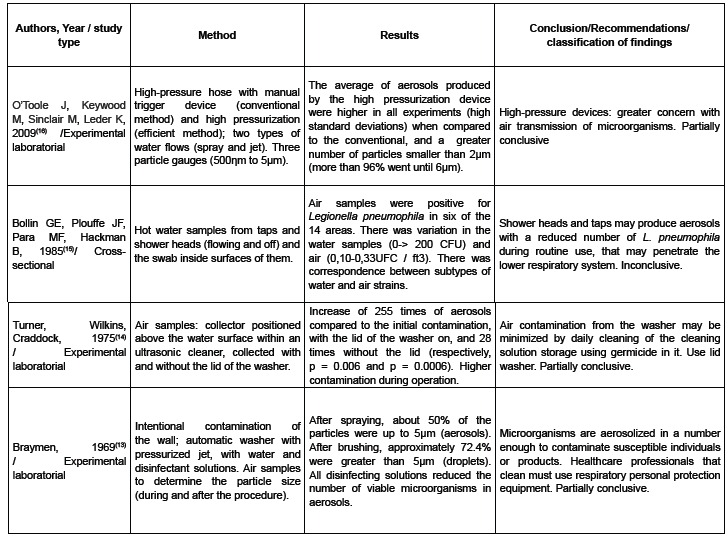
Table of summary results, São Paulo, SP, 2015

The summary of the results is shown in Figure 3.

## Discussion

In the selected technical papers for this review, there is a consensus of the need for negative pressure air in cleaning and decontamination areas of the MD in CSD. This directive corresponds to the norms of the RDC ANVISA 15/2012^7^, where it is stated that in class II CSD a negative differential ambient air pressure (minimum 2,5Pa) should be kept between adjacent areas. However, it was found that both the national and international technical documents do not cite scientific references that prove, with conclusive evidence, the risk of environmental exposure to aerosols for both the MD and for professionals. The classification of CSD as level one, related to the risk of adverse health events by exposure to ambient air^9^, states that this risk is not scientifically proven, but based on expert opinions, which reinforces the need for research to generate data for the support of laws, rules and recommendations. 

As mentioned in the introduction of this study, microorganisms in the air of the CSD environment can be related to two issues: inhalation by professionals and depositing of them on the clean material in the preparation area. Regarding the last issue, considering that the MD will be sterilized before use and that this method was designed to eliminate all forms of microbial life^10^, by theoretical deduction, the possible air contamination of the MD can be considered negligible.

It is noteworthy, in technical documentation, the need for environments with efficient system of negative air pressure having automatic doors, that have all windows and floor sealed, because the opening and closing of a door, associated with the movement of people, immediately reduces the differential air pressure between the areas. In the case of environments with a high circulation of people, as it occurs in the CSD cleaning area, the construction of an anteroom should be considered ^6^ ^,^ ^11^. 

The influence of the movement of doors and people traffic in the negative pressure air efficiency in isolation rooms was demonstrated in the study by Adams, Johnson and Lynch^17^. The air pressure differentials were measured between the room and the anteroom and between the room and the hallway. The average in the aerosol count per m3 where larger when there were greater movement of people and doors, however the aerosol score decreased as the air pressure differential increased. The authors indicate a negative air pressure differential of 20 Pa when there is heavy traffic between areas. In this context, there is a need for routine monitoring of the pressure differential between the areas, for example, by testing with the observation of the direction of smoke flow or specific gauges^18^.

Other parameters shown in Figure 2 relate to the minimum and total number of air changes per hour. The ventilation and air conditioning systems act favorably in controlling infections decreasing the time of exposure to bioaerosols by means of air exchange and differential pressure. In addition, they provide the dilution of the air through supplement and/or exhaustion, reducing the concentration of contaminants; improving air quality by filtration; allowing control of temperature and humidity; and creating air flow patterns^19^. 

The need for negative air pressure in isolation rooms is strongly evidenced in the literature, aiming to reduce the risk of exposure microorganisms transmitted by aerosol infected people to uninfected people (especially other patients or professionals). The advent of diseases such as severe acute respiratory syndrome, avian flu and drug resistant tuberculosis raises concern for health authorities in relation to the isolation of patients^11^ ^,^ ^18^. Also there is no consensus on the number of air changes per hour in isolation rooms^4^.

The release of aerosols also occurs in other sectors of the health system. In the study of Verde and collaborators^20^, the goal was to characterize the air pollution levels in different areas of the hospital (emergency room, surgical ward and operating room). After finishing the procedure, it was detected an increase in the concentration of bacteria in the air. However, the contamination level returned to baseline values (collected in the empty operating room) after cleaning procedures.

Bronchoscopy is a procedure recognized for its potential to generate aerosols, by stimulating coughing patient leading to the contamination of the environment and professionals. In the study by Lavoie and collaborators^21^, although it is not the main goal of the authors, sizes and aerosol concentrations were compared in two bronchoscopy rooms, (one with negative differential air pressure and one without). After statistical analysis, it was shown statistically significant increase in the average concentration of aerosols per m3 in a non-negative differential room during the performance of bronchoscopy, and there was no significant increase in the room with differential. These data shows a greater contamination of the air in rooms without negative pressure, while performing procedures that form aerosols. Despite the concentration of aerosols return to baseline levels more quickly in the room with negative differential air pressure after completion of the procedures, it took fifteen minutes to the bioaerosols levels to return to baseline in both rooms.

The papers included in this review are in the majority originated in previous decades, which demonstrates the need of current research conduction because there were advances in the technology of the equipment used in CSD, for example, in ultrasonic cleaner. The ultrasonic cleaner complement the manual cleaning or are self-cleaning in cleaning the MD with simple conformation depending on the power of ultrasound. They are mainly suitable for complementary cleaning of the complex forming MD and operate on the principle of cavitation (sound waves propagated in an aqueous medium fragmenting rupture or dirt adhered to products)^7^. Despite using the same principle, the equipment currently available are modern, of different dimensions and efficiency when compared to the washer used in the study included in this review^14^, and the fact that they are usually operated capped. A limitation of this study was the definition for the recovery of only one microorganism (*Pseudomonas aeruginosa*) and it was rated as partially conclusive.

In the studies included in this review^13^ ^,^ ^16^, it was shown that using pressurized water cleaning devices generate more aerosol when compared to conventional methods (brushing and low pressurization of water). Considering the guiding question of this review and the fact that these experiments were not performed in a CSD environment they were classified as partially conclusive. On CSD, the pressurized water guns are used for crude dirt cleaning. Compressed air guns are used for drying products with lumen and complex conformation^22^. 

About the size of the particles, it was evidenced in this review that the aerosol generated after the use of pressurized water surface cleaning devices were smaller than 5μm^13^ ^,^ ^16^. In the study of Bollin and collaborators^15^, there are reports that Legionella pneumophila aerosols generated by the tap were larger than the ones in the shower, however, there is no detailed description of the sizes. Particles larger than 10μm are more likely to remain on the surface of the upper airways and not penetrate into the lower lung regions. However, the smaller the particle size, the easier it is its moving until the alveoli^23^. Researchers say less than 5μm aerosols can be easily inhaled and it moves slowly with speed lower than 1m / h^24^.

The size of the aerosol is the factor that most influences in its biological properties and displacement. The permanence of aerosols in ambient air undergoes action of physicochemical processes such as evaporation, interaction with other particles, transportation, gravity, temperature, relative humidity and air currents, among others. There are reports that aerosols remain suspended in the same environment for years^25^. Therefore, there is evidence of air pollution in the CSD cleaning area; however, data on the permanence in the air and displacement of aerosols to adjacent areas is lacking. A limitation of the studies included in this review was the lack of control of environmental factors where the experiments were carried out, which may have underestimated or overestimated the recovery of aerosols.

In a study of this review the contamination of water and air by *Legionella pneumophila* was evaluated and it was identified that the strains detected in aerosols were of the same subtype of the samples of shower water and hot tap water^15^. This study has limitations regarding the collection of data because there has been no standardization of the number of samples, time and air collection instrument, furthermore, the authors collected water samples a week before the air samples, which resulted in its classification as an inconclusive study. 

Environmental contamination by *Legionella pneumophila* aerosols has been reported, mostly from showers, humidifiers and ventilatory support equipment (bag-valve-mask device) rinsed with contaminated water^26^. Infection by these bacteria generates concern especially for immunosuppressed people. In a retrospective study in elderly care institutions^27^, evaluated the contamination of the water supplied to the shower and the occurrence of Pontiac fever symptoms that is a benign form of infection caused by *Legionella pneumophila* and resembles the symptoms of influenza infection. The incidence of Pontiac fever in this population was 0.11 cases/people/year (95% CI 0:07 to 0:15). Of the 32 reported cases, 29 had been exposed to contaminated water. Considering these data, the water of hot water taps contaminated in the CSD can lead to the formation of aerosol with *Legionella pneumophilla.*

In summary of the results of this review, it was found that air contamination by aerosol was higher during the cleaning procedure or while the tap was turned on^13^ ^-^ ^15^, returning to baseline levels after these procedures were completed . These data confirm the need to use respiratory personal protective equipment (PPE) by professionals responsible for cleaning products on CSD. The RDC 15/2002^7^ determines that the reception area and cleaning worker must wear a mask or face shield, goggles, gloves, long sleeve waterproof aprons, ear protection and closed footwear. However, there is no description of the type of mask. Considering the results presented in this review as the size of the aerosols formed, suggests the use of N95 mask (a mask that has the capacity to filter particles <3μm) in the mentioned areas^2^ ^,^ ^13^, although no studies have assessed the risk of disease transmission via aerosol generated in cleaning activities. It is known that, in the chain of transmission of infections, there is need for interaction between the elements: host susceptibility, presence, source, input port and output of the infectious agent and a vehicle of transmission, in addition to the amount of it. Therefore, the risk of CSD employees, or other areas of health services being infected by aerosols vary fundamentally according to the elements of infection transmission chain^19^. 

There were no scientific evidence on occupational diseases related to the cleaning activity in CSD. Greater emphasis is given to accidents with sharp objects, chemical or ergonomic exposure. The use of PPE in CSD is problematic due to the compliance to the use and discomfort reported by the professionals working in this sector^28^. Nurses should the responsibility to raise awareness and motivate the CSD team of the obligation and benefits of using PPE.

Considering the occupational health of the CSD workers, the environment temperature control contributes to the comfort of the professional^10^. The control of this parameter, and the relative humidity is possible through thermo hygrometer installation. However, maintenance of air quality with microbiological approach, based on the values recommended by ASHRAE^19^, it is impractical in the reality of the CSD. In this sector there is no full control of conditions of environmental contamination, as carried out in a controlled production structure, found in the pharmaceutical industry, considering that microorganisms can be released both by professionals (eg, movement of people, sneezing, coughing, expiration, speech) and by the activities carried out there.

Given the above, this review has brought advances in scientific knowledge in the control of air pollution in CSD, providing theoretical foundations for better understanding of the phenomenon studied. It was found that aerosols are inevitably generated during the MD cleaning activities, emphasizing the importance of the use of PPE among cleaning room workers. Because of the risk of the deposition of contaminant particles, this data reinforces the need for decontamination of surfaces touched associated with the hand hygiene of professionals. Considering the aerosols generated during the operation of the ultrasonic cleaner, it is recommended to use it with the lid closed.

## Conclusion

Scientific evidence showed that aerosols are generated during cleaning activities and use of devices found in CSD such as the ultrasonic cleaner and the washer with pressurized water. Although the need for a negative differential ambient air pressure between the cleaning area and adjacent areas in CSD is standardized, no studies evaluating its impact on the dispersion of aerosols were found, which could compromise the safety of the material in the preparation room and the health of the cleaning room professionals and the ones that circulate in the adjacent areas.

The studies have not provided information regarding the occupational health of workers in the surrounding areas, but there are strong indications for the use of the N95 mask by cleaning area workers. It demonstrates the need to conduct research on the occurrence of occupational diseases in the workers of CSD.

## References

[1] Morawska L (2006). Droplet fate in indoor environments, or can we prevent the spread of
infection. Indoor Air.

[2] Agência Nacional de Vigilância Sanitária (BR) (2000). Curso Ba´sico de Controle de Infecc¸a~o Hospitalar - Caderno C Me´todos de
Protec¸a~o Anti-Infecciosa.

[3] Siegel JD, Rhinehart E, Jackson M, Chiarello L (2007). Healthcare Infection Control Practices Advisory Committee. 2007 Guideline for
Isolation Precautions: Preventing Transmission of Infectious Agents in Healthcare
Settings.

[4] Hobday RA, Dancer SJ (2013). Roles of sunlight and natural ventilation for controlling infection
historical and current perspectives. J Hosp Infect.

[5] Gehanno JF, Louvel A, Nouvellon M, Caillard JF, Pestel-Caron M (2009). Aerial dispersal of meticillin-resistant Staphylococcus aureus in
hospital rooms by infected or colonised patients. J Hosp Infect.

[6] American Society of Heating.Refrigerating.and Air-Conditioning
Engineers (2011). Health-Care Facilities. 2011 ASHRAE Handbook. Heating, Ventilating and Air Conditioning Application.
SI Edition.

[7] Resolução - RDC Nº 15, de 15 de março de 2012 (BR) (2012). Dispõe sobre requisitos de boas práticas para o processamento de produtos
para saúde.

[8] Hutton B, Salanti G, Caldwell DM, Chaimani A, Schmid CH, Cameron C (2015). The PRISMA Extension Statement for Reporting of Systematic Reviews
Incorporating Network Meta-analyses of Health Care Interventions Checklist and
Explanations. Ann Intern Med.

[9] Associação Brasileira de Normas Técnicas (2005). NBR 7256: 2005, de 30 de março de 2005. 2005. Dispõe sobre o Tratamento de ar
em estabelecimentos assistenciais de saúde (EAS) - Requisitos para projeto e
execução das instalações.

[10] Association for the Advancement of Medical Instrumentation (2006). Comprehensive guide to steam sterilization and sterility assurance in health
care facilities.

[11] Sehulster LM, Chinn RYW, Arduino MJ, Carpenter J, Donlan R, Ashford D (2003). Healthcare Infection Control Practices Advisory Committee. Guidelines for
Environmental Infection Control in Health-Care Facilities. Recommendations of CDC
and the Healthcare Infection Control Practices Advisory Committee
(HICPAC).

[12] American Institute of Architects Academy of Architecture for
Healthcare (2001). Guidelines for design and construction of hospital and health care
facilities.

[13] Braymen DT (1969). Survival of micro-organisms in aerosols produced in cleaning and
disinfecting. Public Health Rep.

[14] Turner AG, Wilkins JR, Craddock JG (1975). Bacterial aerosolization from an ultrasonic cleaner. J Clin Microbiol.

[15] Bollin GE, Plouffe JF, Para MF, Hackman B (1985). Aerosols containing Legionella pneumophila generated by shower heads
and hot-water faucets. Appl Environ Microbiol.

[16] O'Toole J, Keywood M, Sinclair M, Leder K. (2009). Risk in the mist Deriving data to quantify microbial health risks
associated with aerosol generation by water-efficient devices during typical
domestic water-using activities. Water Sci Technol.

[17] Adams NJ, Johnson DL, Lynch RA (2011). The effect of pressure differential and care provider movement on
airborne infectious isolation room containment effectiveness. Am J Infect Control.

[18] Walker JT, Hoffman P, Bennett AM, Vos MC, Thomas M, Tomlinson N (2007). Hospital and community acquired infection and the built environment -
design and testing of infection control rooms. J Hosp Infect.

[19] American Society of Heating.Refrigerating.and Air-Conditioning
Engineers (2013). HVAC design manual for hospital and clinics.

[20] Verde SC, Almeida SM, Matos J, Guerreiro D, Meneses M, Faria T (2015). Microbiological assessment of indoor air quality at different hospital
sites. Res Microbiol.

[21] Lavoie J, Marchand G, Cloutier Y, Hallé S, Nadeau S, Duchaine C (2015). Evaluation of bioaerosol exposures during hospital bronchoscopy
examinations. Environ Sci Process Impacts.

[22] Graziano KU, Lacerda RA, Turrini RNT, Bruna CQM, Silva CPR, Schmitt C (2009). Indicadores de avaliação do processamento de artigos
odonto-médico-hospitalares: elaboração e validação. Rev Esc Enferm USP.

[23] Gralton J, Tovey E, McLaws ML, Rawlinson WD (2011). The role of particle size in aerosolised pathogen transmission a
review. J Infect.

[24] Qian H, Li Y, Nielsen PV, Hyldgaard CE, Wong TW, Chwang AT (2006). Dispersion of exhaled droplet nuclei in a two-bed hospital ward with
three different ventilation systems. Indoor Air.

[25] Cole EC, Cook CE (1998). Characterization of infectious aerosols in health care facilities an
aid to effective engineering controls and preventive strategies. Am J Infect Control.

[26] Woo AH, Yu VL, Goetz A (1986). Potential in-hospital modes of transmission of Legionella pneumophila
Demonstration experiments for dissemination by showers, humidifiers, and rinsing
of ventilation bag apparatus. Am J Med.

[27] Bauer M, Mathieu L, Deloge-Abarkan M, Remen T, Tossa P, Hartemann P (2008). Legionella bacteria in shower aerosols increase the risk of Pontiac
fever among older people in retirement homes. J Epidemiol Commun Health.

[28] Ribeiro RP, Vianna LAC (2012). Uso dos equipamentos de proteção individual entre trabalhadores das
centrais de material e esterilização. Cienc Cuid Saude.

